# Sampling strategies for accurate computational inferences of gametic phase across highly polymorphic major histocompatibility complex loci

**DOI:** 10.1186/1756-0500-4-151

**Published:** 2011-05-26

**Authors:** Miguel Alcaide, Airam Rodríguez, Juan J Negro

**Affiliations:** 1 Department of Organismic and Evolutionary Biology, Harvard University, Cambridge, MA 02138, USA; 2 Department of Evolutionary Ecology, Estación Biológica de Doñana CSIC, Avda. Américo Vespucio s/n, 41092 Seville, Spain

## Abstract

**Background:**

Genes of the Major Histocompatibility Complex (MHC) are very popular genetic markers among evolutionary biologists because of their potential role in pathogen confrontation and sexual selection. However, MHC genotyping still remains challenging and time-consuming in spite of substantial methodological advances. Although computational haplotype inference has brought into focus interesting alternatives, high heterozygosity, extensive genetic variation and population admixture are known to cause inaccuracies. We have investigated the role of sample size, genetic polymorphism and genetic structuring on the performance of the popular Bayesian PHASE algorithm. To cover this aim, we took advantage of a large database of known genotypes (using traditional laboratory-based techniques) at single MHC class I (N = 56 individuals and 50 alleles) and MHC class II B (N = 103 individuals and 62 alleles) loci in the lesser kestrel *Falco naumanni*.

**Findings:**

Analyses carried out over real MHC genotypes showed that the accuracy of gametic phase reconstruction improved with sample size as a result of the reduction in the allele to individual ratio. We then simulated different data sets introducing variations in this parameter to define an optimal ratio.

**Conclusions:**

Our results demonstrate a critical influence of the allele to individual ratio on PHASE performance. We found that a minimum allele to individual ratio (1:2) yielded 100% accuracy for both MHC loci. Sampling effort is therefore a crucial step to obtain reliable MHC haplotype reconstructions and must be accomplished accordingly to the degree of MHC polymorphism. We expect our findings provide a foothold into the design of straightforward and cost-effective genotyping strategies of those MHC loci from which locus-specific primers are available.

## Background

Highly polymorphic genes of the Major Histocompatibility Complex (MHC) have become very popular molecular markers among evolutionary biologists because of their traditional consideration as 'good genes' involved in pathogen resistance and sexual selection (reviewed by [1,2]). Despite a plethora of new methods and technical advances (reviewed by [3]), MHC genotyping still remains challenging and time-consuming. Recently, Bayesian computational inference of gametic phase coupled to Sanger sequencing of PCR amplicons has emerged as a promising alternative [4-7]. These in-silico methods permit researchers to infer how multiple segregating sites are distributed within the same chromosome and are believed to provide haplotype information in a more straightforward and cost-effective way than laboratory-based methods such as cloning, non-denaturing gel electrophoresis and others (reviewed in [3]). Even though extremely variable MHC loci subjected to the effects of natural selection violate several assumptions of the underlying neutral coalescent theory [5], computer packages such as PHASE have shown to perform admirably in many cases [7-10]. The current version of PHASE, that provides a biologically realistic prior for the distribution of haplotypic frequencies [6], has become one of the most preferred options among evolutionary biologist because of its good performance and the possibility to deal with gaps and polymorphic sites with up to four segregating sites. The accuracy of gametic phase inference has shown to be, however, very sensitive to high heterozygosity, large numbers of alleles and population admixture [e.g. [8]]. The two first factors are particularly common among MHC genes, a fact that can explain low success rates for particular data sets [8]. In spite of the cost and sample manipulation advantages put forward by these approaches [reviewed in [3]], only a few studies (e.g. [8,10]) have addressed in detail the relative role of different parameters on PHASE performance when working with highly polymorphic and recombining MHC loci usually exhibiting the genetic hallmarks of balancing and positive selection (i.e. excess of heterozygous sites and non-synonymous substitutions). In this study, we have taken advantage of a large database of MHC class I and class II genotypes built from traditional molecular cloning in the lesser kestrel *Falco naumanni*. Our mains goals were i) test the performance of analytical approaches to haplotype inference in the kestrel MHC, and ii) evaluate the influence of sample size, genetic polymorphism and genetic structure on the accuracy of computational approaches dealing with phase-unknown diploid genotypes.

## Methods

The MHC of the lesser kestrel is well suited for this study because of the specific amplification via the polymerase chain reaction (PCR) of single, highly polymorphic and positively selected MHC class I (exon 3) and MHC class II B (exon 2) loci [11,12]. Both loci are 270 base pairs in length and encode for part of the antigen-binding region of MHC class I and MHC class II molecules, respectively. Heterozygosity has been shown to be extremely large in natural populations at both loci (> 90%, [13,14]). A large proportion of the MHC alleles used in this study were isolated during previous studies and many others are derived from ongoing research [[11-14], authors unpublished data, see additional file 1]. The handling and sampling of the birds was done in accordance with Spanish laws concerning animal welfare, and under permission of the different National Governments.

We created two data sets, one for each particular MHC locus. Overall, we gathered the known genotypes of 56 heterozygous birds at the MHC class I locus and 103 heterozygous individuals at the MHC class II B locus. Even though homozygous individuals at both loci have been reported we decided to exclude them from our data sets as a means to create the most challenging scenario during the evaluation of the performance of the PHASE algorithm. The two data sets encompassed 50 MHC class I and 62 MHC class II alleles, respectively (see Table 1 and additional files 1 and 2). For the MHC class I data set, only four and two alleles, respectively, showed frequencies beyond 5% and 10%. For the MHC class II data set, only seven and three alleles, respectively, showed frequencies higher than 5% and 10%. The two data sets also represented different degrees of genetic structuring. In the case of the MHC class I, individual genotypes were obtained from birds captured in Spain, France, Italy, Greece and Israel and restrictions in gene flow are thus expected (see [13]). Individuals of the MHC class II data set were exclusively sampled from Spain, which can be essentially considered as a panmictic population according to both neutral and adaptive genetic data [13,15]. We created different sample subsets containing 8, 15, 30 or 45 individuals from the MHC class I data set. In the case of the MHC class II, sample subsets were composed of 8, 15, 30, 45, 60 or 75 individuals. Five groups of individuals were randomly sub-sampled for each sample size.

**Table 1:** Polymorphisms statistics at the kestrel MHC class I and class II data sets used in this study.

Locus	Na	S	Eta	π	*k*
MHC class I	50	37	41	0.030	8.45
MHC class II	62	60	75	0.078	21.04

This table compiles the number of alleles (Na), the number of variable sites (S), the total number of mutations (Eta), mean nucleotide diversity per site (π) and the average number of nucleotide differences between alleles (*k*).

The knowledge of the real genotypes beforehand permitted us to generate those ambiguous DNA sequences resulting from the overlapping of the two alleles isolated per individual at each MHC locus (see additional file 2). These consensus DNA sequences were generated using the software BioEdit [16]. With this information, we performed a reverse approach through which analytical approaches relying on ambiguous diploid data would be validated with respect to the genotypes inferred using traditional laboratory-based techniques. Bayesian computational inference of MHC gametic phase was performed using the popular, user-friendly PHASE module implemented in the software DNAsp ver 5.0 [17]. Calculations were carried out over 1,000 iterations, 10 thinning interval and 1,000 burn-in iterations and considering a model that accounted for recombination. All the advanced options available for the algorithm were settled as default. PHASE accuracy was measured as the percentage of correctly assigned alleles. We concluded that the two alleles at each locus were correctly inferred when all nucleotide positions matched perfectly to those previously revealed by laboratory-based methods. To verify the identity of each allele, we took advantage of the output window provided by default by the software DNAsp 5.0 and we exported the alignment as a FASTA file subsequently handled in BioEdit.

## Results

**Figure 1:**
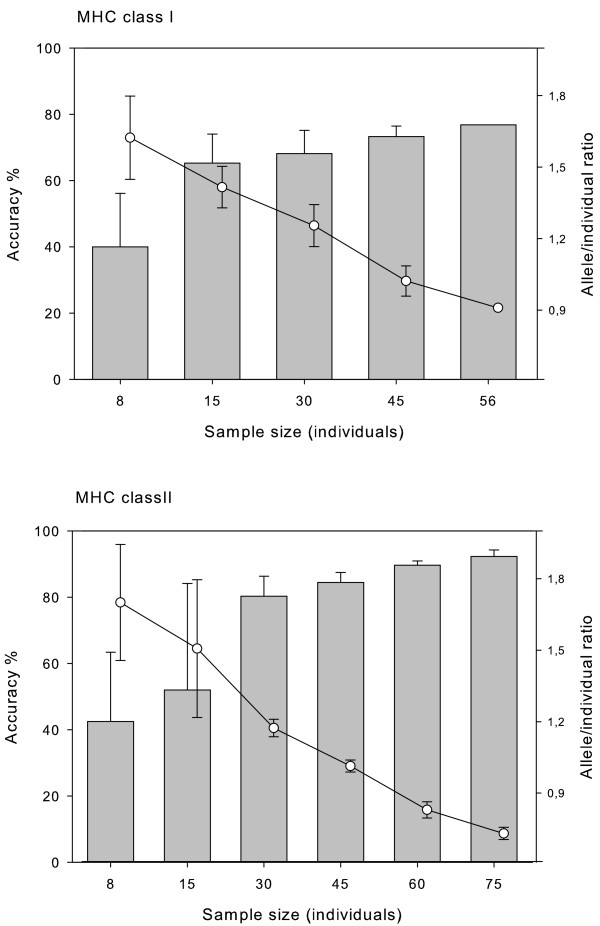
**Influence of the number of individuals analysed on the performance (percentage of alleles correctly assigned; bars go with primary Y-axis) of Bayesian reconstructions of MHC haplotypes using the PHASE algorithm and the allele-to-individual ratio (open circles go with secondary Y-axis)**. Standard deviations for each parameter are indicated.

**Figure 2:**
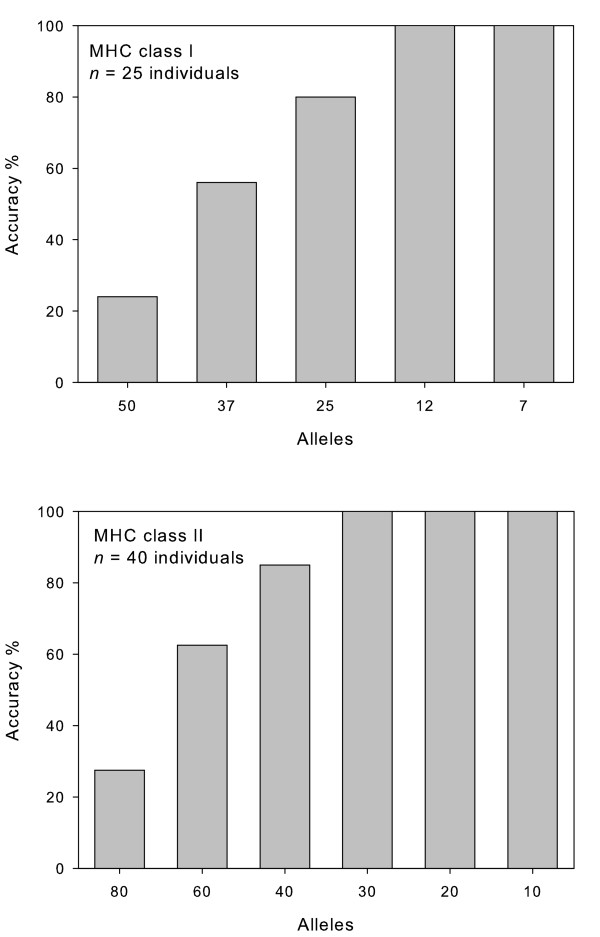
**Influence of the allele-to-individual ratio on the performance of Bayesian reconstructions of MHC haplotypes using the PHASE algorithm**. We altered the number of alleles for a sample size of 25 individuals for the MHC class I and 40 individuals for the MHC class II data sets. Standard deviations for each parameter are indicated.

Our results show a remarkable influence of sample size on the accuracy of haplotypic inference using PHASE (Figure 1). For both MHC loci, average accuracy improved along with sample size. The number of alleles not correctly inferred was proportional to the number of genotypes. This is due to fact that when PHASE failed to infer one of the two alleles from a given genotype it incorrectly inferred the sequence of the other allele as well (i.e. one or a few segregating sites where switched between the two alleles). Overall, PHASE errors were related to the incorrect calling of one or a few segregating sites, and at least, PHASE seemed to do rather well when inferring the allelic lineage. The increase of PHASE accuracy along with sample size can be attributed to the reduction in the ratio between the number of alleles occurring in the sample set and the number of individuals comprising that particular sample set. To get deeper insights about the influence of the allele to individual ratio, we created simulated data that introduced variations in this parameter. In these simulations, we altered the allele-to-individual ratio for a sample size of 25 individuals and 40 individuals for the MHC class I and class II locus, respectively. The simulated genotypes were heterozygous in all cases and we tried to distribute allele frequencies as equally as possible. Only in the case of the simulation of seven class I alleles (see Figure 2) we repeated 4 out the 25 heterozygous genotypes used in the same sample set. In the remaining cases, the number of possible combinations of alleles in heterozygous form was larger than sample size (i.e. N = 25 and N = 40 for the MHC class I and class II data set, respectively). We added 15 MHC class II B alleles isolated during previous studies [13,14] in order to gather the 80 alleles needed for the 2:1 allele individual ratio. The manipulation of the allele-to-individual ratio had a dramatic influence on PHASE performance (Figure 2). For instance, the accuracy of computational inferences of MHC haplotypes was very poor when the number of alleles was twice than that of individuals. Nonetheless, the performance of PHASE consistently increased along with the reduction of the allele to individual ratio. From the comparison between the two MHC data sets, and regardless of the degree of genetic structuring within the geographic area individuals were sampled from, we suggest a ratio allele to individual starting at (1:2).

The main objective of this study was to provide useful information regarding the number of individuals to be sampled, given a particular degree of genetic polymorphism, to computationally infer the gametic phase of MHC genes with reliability. Starting from a "worst-case" scenario similar to that used in our simulations (i.e. no occurrence of homozygous individuals and with homogenous distributions of allele frequencies), we recommend a first exploratory view of 25-30 individuals. Although PHASE can miscall nucleotides during the reconstruction of haplotypes, our experience suggests that the overall number of alleles inferred is not very different from the actual number. Depending on the number of alleles inferred by PHASE, researchers might add more individuals until the allele to individual ratio reaches at least the 1:2 threshold. Sampling strategies must therefore be designed according to the extent of MHC polymorphisms found within a particular study population. Hopefully, researchers might find homozygous genotypes or genotypes comprised by alleles just differing in one or a few nucleotides during sampling. This might be indeed very useful regarding the verification of the set of inferred alleles. It is also advisable to ground-truth the data set by performing molecular cloning in a selected number of individuals. Molecular cloning, however, is extremely prone to report false polymorphisms and therefore, it is important to contrast cloned alleles with direct sequencing chromatograms. Special caveats should be considered in the case of synonymous diploid genotypes (i.e. different combinations of alleles can generate the same direct sequencing chromatogram). However, careful examination of our allele repertoire suggests that these cases are rare in kestrels (< 1% of possible genotypes). The additional aid of technologies such as conformational polymorphism analyses (e.g. [18]) may nonetheless become very useful to resolve these particular cases. Researchers must pay special attention to generate high-quality direct sequencing chromatograms to minimize the risk of miscall double peaks. In this respect, the performance and location of sequencing primers as well as bi-directional sequencing must be carefully addressed. Finally, it is important to bear in mind that these approaches can only be achieved when locus-specific primers are available [[19,20], this study]. That said, our better genomic knowledge of the MHC in both model and non-model species (e.g. [21,22]) forecasts an encouraging future in this respect.

## Competing interests

The authors declare that they have no competing interests.

## Authors' Contributions

MA and AR contributed equally to this work. They designed and carried out the simulations, performed the sequence alignment, and drafted the manuscript. JJN conceived the study, revised the manuscript and was responsible for the research grant that funded this study. All authors read and approved the final manuscript.

## Supplementary Material

Additional file 1 **MHC class I and MHC class II B genotypes**. Genotypes resolved by traditional laboratory-based methods during previous studies [11-14] and ongoing research by the authors. GenBank accession numbers for the MHC alleles of the lesser kestrel *Falco naumanni *are shown. Click here for file

Additional file 2 **Original data set of unphased MHC class I and II genotypes**. Simulations contain 8, 15, 30 or 45 individuals from the MHC class I, and 8, 15, 30, 45, 60 or 75 individuals from the MHC class II. Five replicates for each sample size were created, sub-sampling individuals randomly. Click here for file

## References

[B1] Sommer S The importance of immune gene variability (MHC) in evolutionary ecology and conservation Front Zool 2005 2 16 10.1186/1742-9994-2-16 16242022 PMC1282567

[B2] Piertney S Oliver M The evolutionary ecology of the major histocompatibility complex Heredity 2006 96 7 21 16094301 10.1038/sj.hdy.6800724

[B3] Babik W Methods for MHC genotyping in non model vertebrates Mol Ecol Res 2010 10 237 251 10.1111/j.1755-0998.2009.02788.x 21565019

[B4] Niu TH Qin ZHS Xu XP Liu JS Bayesian haplotype inference for multiple linked single-nucleotide polymorphisms Am J Hum Genet 2002 70 157 169 10.1086/338446 11741196 PMC448439

[B5] Stephens M Smith NJ Donnelly P A new statistical method for haplotype reconstruction from population data Am J Hum Genet 2001 68 978 989 10.1086/319501 11254454 PMC1275651

[B6] Stephens M Donnelly P A comparison of Bayesian methods for haplotype reconstruction from population genotype data Am J Hum Genet 2003 73 1162 1169 10.1086/379378 14574645 PMC1180495

[B7] Harrigan RJ Mazza ME Sorenson MD Computation versus cloning: evaluation of two methods for haplotype determination Mol Ecol Res 2008 8 1239 1248 10.1111/j.1755-0998.2008.02241.x 21586011

[B8] Bos DH Turner SM Dewoody JA Haplotype inference from diploid sequence data: evaluating performance using non-neutral MHC sequences Hereditas 2007 144 228 234 10.1111/j.2007.0018-0661.01994.x 18215245

[B9] Garrick RC Sunnucks P Dyer RJ Nuclear gene phylogeography usingh PHASE: dealing with unresolved genotypes, lost alleles, and systematic bias in parameter estimation BMC Evol Biol 2010 10 118 10.1186/1471-2148-10-118 20429950 PMC2880299

[B10] Bettencourt BF Santos MR Fialho RN Couto AR Peixoto MJ Pinheiro JP Evaluation of two methods for computational HLA haplotype inference using a real data set BMC Bioinformatics 2008 9 68 10.1186/1471-2105-9-68 18230173 PMC2268655

[B11] Alcaide M Edwards SV Negro JJ Characterization, polymorphism, and evolution of MHC class II B genes in birds of prey J Mol Evol 2007 65 541 554 10.1007/s00239-007-9033-9 17925996

[B12] Alcaide M Edwards SV Cadahia L Negro JJ MHC class I genes of birds of prey: isolation, polymorphism and diversifying selection Conserv Genet 2009 10 1349 1355 10.1007/s10592-008-9653-7

[B13] Alcaide M Edwards SV Negro JJ Serrano D Tella JL Extensive polymorphism and geographical variation at a positively selected MHC class II B gene of the lesser kestrel (*Falco naumanni*) Mol Ecol 2008 17 2652 2665 10.1111/j.1365-294X.2008.03791.x 18489548

[B14] Alcaide M Lemus JA Blanco G Tella JL Serrano D Negro JJ Rodríguez A García-Montijano M MHC diversity and differential exposure to pathogens in kestrels (Aves: Falconidae) Mol Ecol 2010 19 691 705 10.1111/j.1365-294X.2009.04507.x 20074317

[B15] Alcaide M Serrano D Tella JL Negro JJ Strong philopatry derived from capture-recapture records does not lead to fine-scale genetic differentiation in lesser kestrels J Anim Ecol 2009 78 468 475 10.1111/j.1365-2656.2008.01493.x 19054221

[B16] Hall TA BioEdit: a user-friendly biological sequence alignment editor and analysis program for Windows 95/98/NT Nucl Acids Symp Ser 1999 41 95 98

[B17] Librado P Rozas J DnaSP v5: a software for comprehensive analysis of DNA polymorphism data Bioinformatics 2009 25 1451 1452 10.1093/bioinformatics/btp187 19346325

[B18] Alcaide M Lopez L Tanferna A Blas J Sergio F Hiraldo F Simultaneous analysis of multiple PCR amplicons enhances capillary SSCP discrimination of MHC alleles Electrophoresis 2010 31 1353 1356 10.1002/elps.200900709 20358545

[B19] Bettinotti MP Hadzikadic L Ruppe E Dhillon G Stroncek DS Marincola FM New HLA-A, -B, and -C locus-specific primers for PCR amplification from cDNA: application in clinical immunology J Immunol Methods 2003 279 143 148 10.1016/S0022-1759(03)00233-3 12969555

[B20] Hughes CR Miles S Walbroehl JM Support for the minimal essential MHC hypothesis: a parrot with a single, highly polymorphic MHC class II B gene Immunogenetics 2008 60 219 231 10.1007/s00251-008-0287-1 18431567

[B21] Worley K Gillingham M Jensen P Kennedy LJ Pizzari T Kaufman J Richardson D Single locus typing of MHC class I and class II B loci in a population of red jungle fowl Immunogenetics 2008 60 233 247 10.1007/s00251-008-0288-0 18389232

[B22] Cloutier A Mills JA Baker AJ Characterization and locus-specific typing of MHC class I genes in the red-billed gull (*Larus scolopinus*) provides evidence for major, minor, and nonclassical loci Immunogenetics 2011 63 377 394 10.1007/s00251-011-0516-x 21327606

