# Supplementary material for: Sampling strategies for accurate computational inferences of gametic phase across highly polymorphic major histocompatibility complex loci
Source: BMC Res Notes. 2011 May 26;4:151. doi: 10.1186/1756-0500-4-151 (PMC3126723; doi:10.1186/1756-0500-4-151)
Supplement: Additional file 1 — MHC class I and MHC class II B genotypes. Genotypes resolved by traditional laboratory-based methods during previous studies [11-14] and ongoing research by the authors. GenBank accession numbers for the MHC alleles of the lesser kestrel Falco naumanni are shown. [file 1756-0500-4-151-S1.DOC]

**Additional file 1**

File format: DOC

Title: MHC class I and MHC class II B genotypes resolved by traditional laboratory-based methods during previous studies [11-14] and ongoing research by the authors. GenBank accession numbers for the MHC alleles of the lesser kestrel *Falco naumanni* are shown.

| **Genotype** | **MHC Class I** | | **MHC Class II** | |
| --- | --- | --- | --- | --- |
| 1 | *Fana10* | *Fana11* | *Fana35* | *Fana36* |
| 2 | *Fana2* | *Fana13* | *Fana62* | *Fana83* |
| 3 | *Fana2* | *Fana11* | *Fana1* | *Fana19* |
| 4 | *Fana8* | *Fana18* | *Fana19* | *Fana41* |
| 5 | *Fana12* | *Fana33* | *Fana29* | *Fana40* |
| 6 | *Fana4* | *Fana11* | *Fana4* | *Fana12* |
| 7 | *Fana11* | *Fana19* | *Fana2* | *Fana91* |
| 8 | *Fana14* | *Fana17* | *Fana2* | *Fana71* |
| 9 | *Fana2* | *Fana41* | *Fana17* | *Fana7* |
| 10 | *Fana20* | *Fana47* | *Fana1* | *Fana2* |
| 11 | *Fana1* | *Fana35* | *Fana34* | *Fana35* |
| 12 | *Fana11* | *Fana13* | *Fana2* | *Fana26* |
| 13 | *Fana10* | *Fana29* | *Fana3* | *Fana79* |
| 14 | *Fana2* | *Fana34* | *Fana60* | *Fana10* |
| 15 | *Fana4* | *Fana10* | *Fana19* | *Fana47* |
| 16 | *Fana6* | *Fana11* | *Fana2* | *Fana8* |
| 17 | *Fana2* | *Fana16* | *Fana1* | *Fana33* |
| 18 | *Fana3* | *Fana10* | *Fana3* | *Fana91* |
| 19 | *Fana11* | *Fana52* | *Fana26* | *Fana29* |
| 20 | *Fana9* | *Fana19* | *Fana60* | *Fana36* |
| 21 | *Fana2* | *Fana35* | *Fana8* | *Fana39* |
| 22 | *Fana6* | *Fana36* | *Fana39* | *Fana68* |
| 23 | *Fana11* | *Fana59* | *Fana2* | *Fana19* |
| 24 | *Fana2* | *Fana48* | *Fana11* | *Fana12* |
| 25 | *Fana27* | *Fana60* | *Fana12* | *Fana19* |
| 26 | *Fana11* | *Fana20* | *Fana2* | *Fana7* |
| 27 | *Fana15* | *Fana38* | *Fana40* | *Fana31* |
| 28 | *Fana6* | *Fana40* | *Fana2* | *Fana83* |
| 29 | *Fana9* | *Fana42* | *Fana2* | *Fana65* |
| 30 | *Fana2* | *Fana24* | *Fana1* | *Fana8* |
| 31 | *Fana7* | *Fana10* | *Fana111* | *Fana82* |
| 32 | *Fana17* | *Fana20* | *Fana19* | *Fana48* |
| 33 | *Fana24* | *Fana25* | *Fana26* | *Fana82* |
| 34 | *Fana1* | *Fana5* | *Fana79* | *Fana80* |
| 35 | *Fana2* | *Fana6* | *Fana29* | *Fana51* |
| 36 | *Fana7* | *Fana11* | *Fana31* | *Fana50* |
| 37 | *Fana13* | *Fana28* | *Fana2* | *Fana87* |
| 38 | *Fana38* | *Fana57* | *Fana3* | *Fana7* |
| 39 | *Fana2* | *Fana20* | *Fana2* | *Fana40* |
| 40 | *Fana34* | *Fana62* | *Fana7* | *Fana21* |
| 41 | *Fana2* | *Fana27* | *Fana1* | *Fana3* |
| 42 | *Fana2* | *Fana45* | *Fana1* | *Fana85* |
| 43 | *Fana20* | *Fana35* | *Fana9* | *Fana1* |
| 44 | *Fana7* | *Fana19* | *Fana19* | *Fana42* |
| 45 | *Fana19* | *Fana63* | *Fana36* | *Fana72* |
| 46 | *Fana11* | *Fana46* | *Fana3* | *Fana35* |
| 47 | *Fana17* | *Fana47* | *Fana2* | *Fana53* |
| 48 | *Fana32* | *Fana47* | *Fana2* | *Fana63* |
| 49 | *Fana2* | *Fana7* | *Fana29* | *Fana36* |
| 50 | *Fana20* | *Fana50* | *Fana1* | *Fana27* |
| 51 | *Fana11* | *Fana35* | *Fana19* | *Fana67* |
| 52 | *Fana20* | *Fana22* | *Fana19* | *Fana52* |
| 53 | *Fana11* | *Fana23* | *Fana19* | *Fana68* |
| 54 | *Fana10* | *Fana30* | *Fana19* | *Fana87* |
| 55 | *Fana27* | *Fana31* | *Fana23* | *Fana2* |
| 56 | *Fana6* | *Fana39* | *Fana12* | *Fana37* |
| 57 |  |  | *Fana2* | *Fana4* |
| 58 |  |  | *Fana1* | *Fana72* |
| 59 |  |  | *Fana1* | *Fana12* |
| 60 |  |  | *Fana12* | *Fana33* |
| 61 |  |  | *Fana82* | *Fana83* |
| 62 |  |  | *Fana2* | *Fana37* |
| 63 |  |  | *Fana8* | *Fana19* |
| 64 |  |  | *Fana68* | *Fana75* |
| 65 |  |  | *Fana9* | *Fana36* |
| 66 |  |  | *Fana29* | *Fana107* |
| 67 |  |  | *Fana2* | *Fana89* |
| 68 |  |  | *Fana111* | *Fana2* |
| 69 |  |  | *Fana2* | *Fana57* |
| 70 |  |  | *Fana1* | *Fana36* |
| 71 |  |  | *Fana2* | *Fana3* |
| 72 |  |  | *Fana31* | *Fana62* |
| 73 |  |  | *Fana36* | *Fana83* |
| 74 |  |  | *Fana47* | *Fana60* |
| 75 |  |  | *Fana1* | *Fana34* |
| 76 |  |  | *Fana35* | *Fana1* |
| 77 |  |  | *Fana8* | *Fana10* |
| 78 |  |  | *Fana81* | *Fana94* |
| 79 |  |  | *Fana2* | *Fana10* |
| 80 |  |  | *Fana10* | *Fana32* |
| 81 |  |  | *Fana1* | *Fana21* |
| 82 |  |  | *Fana19* | *Fana20* |
| 83 |  |  | *Fana32* | *Fana70* |
| 84 |  |  | *Fana2* | *Fana31* |
| 85 |  |  | *Fana1* | *Fana18* |
| 86 |  |  | *Fana9* | *Fana17* |
| 87 |  |  | *Fana19* | *Fana83* |
| 88 |  |  | *Fana1* | *Fana25* |
| 89 |  |  | *Fana4* | *Fana90* |
| 90 |  |  | *Fana2* | *Fana60* |
| 91 |  |  | *Fana34* | *Fana41* |
| 92 |  |  | *Fana31* | *Fana68* |
| 93 |  |  | *Fana87* | *Fana109* |
| 94 |  |  | *Fana19* | *Fana34* |
| 95 |  |  | *Fana52* | *Fana26* |
| 96 |  |  | *Fana8* | *Fana40* |
| 97 |  |  | *Fana19* | *Fana62* |
| 98 |  |  | *Fana2* | *Fana36* |
| 99 |  |  | *Fana2* | *Fana62* |
| 100 |  |  | *Fana82* | *Fana2* |
| 101 |  |  | *Fana3* | *Fana82* |
| 102 |  |  | *Fana19* | *Fana36* |
| 103 |  |  | *Fana111* | *Fana57* |

**Genbank accession numbers**

**MHC class I locus**

*Fana*1: EU120671

*Fana*2: EU120672

*Fana*3: EU120675

*Fana*4: EU120667

*Fana*5: JF831086

*Fana*6: EU120676

*Fana*7: EU120668

*Fana*8: EU120669

*Fana*9: EU120670

*Fana*10: EU120665

*Fana*11: EU120664

*Fana*12: EU120674

*Fana*13: EU120666

*Fana*14: JF831087

*Fana*15: EU120673

*Fana*16: EU120677

*Fana*17: EU120678

*Fana*18: EU120679

*Fana*19: JF831088

*Fana*20: JF831089

*Fana*22: JF831090

*Fana*23: JF831091

*Fana*24: JF831092

*Fana*25: JF831093

*Fana*27: JF831094

*Fana*28: JF831095

*Fana*29: JF831096

*Fana*30: JF831097

*Fana*31: JF831098

*Fana*32: JF831101

*Fana*33: JF831099

*Fana*34: JF831102

*Fana*35: JF831100

*Fana*36: JF831105

*Fana*38: JF831104

*Fana*39: JF831103

*Fana*40: JF831110

*Fana*41: JF831108

*Fana*42: JF831111

*Fana*45: JF831109

*Fana*46: JF831112

*Fana*47: JF831107

*Fana*48: JF831113

*Fana*50: JF831114

*Fana*52: JF831115

*Fana*57: JF831116

*Fana*59: JF831117

*Fana*60: JF831118

*Fana*62: JF831119

*Fana*63: JF831120

**MHC class II B locus**

*Fana*1: EF370839

*Fana*2: EF370840

*Fana*3: EF370841

*Fana*4: EU107670

*Fana*7: EF370855

*Fana*8: EF370851

*Fana*9: EF370850

*Fana*10: EF370842

*Fana*11: EF370848

*Fana*12: EF370849

*Fana*17: EU107729

*Fana*18: EF370852

*Fana*19: EF370854

*Fana*20: EF370853

*Fana*21: EF370846

*Fana*23: EU107668

*Fana*25: EU107715

*Fana*26: EU107703

*Fana*27: EF370847

*Fana*29: EF370860

*Fana*31: EF370858

*Fana*32: EF370845

*Fana*33: EF370844

*Fana*34: EU107671

*Fana*35: EU107704

*Fana*36: EF370861

*Fana*37: EU107722

*Fana*39: EU107743

*Fana*40: EU107734

*Fana*41: EU107721

*Fana*42: EU107682

*Fana*47: EU107680

*Fana*48: EU107733

*Fana*50: EU107726

*Fana*51: EU107725

*Fana*52: EU107744

*Fana*53: EF370864

*Fana*57: EU107707

*Fana*60: EU107740

*Fana*62: EU107741

*Fana*63: EU107709

*Fana*65: EU107686

*Fana*67: EU107667

*Fana*68: EF370843

*Fana*70: EF370859

*Fana*71: EF370863

*Fana*72: EU107681

*Fana*75: EU107687

*Fana*79: EU107739

*Fana*80: EU107695

*Fana*81: EU107730

*Fana*82: EF370856

*Fana*83: EF370857

*Fana*85: EF370862

*Fana*87: EU107692

*Fana*89: EU107728

*Fana*90: EU107711

*Fana*91: EU107693

*Fana*94: EU107677

*Fana*107: EU107738

*Fana*109: EU107672

*Fana*111: HQ402919
